# Healthy food and dietary patterns: a cross-sectional study in Riyadh, Saudi Arabia

**DOI:** 10.3389/fpubh.2025.1716376

**Published:** 2026-01-09

**Authors:** Noara Alhusseini, Maryam Sajjad, Heba Rahman, Hamna Naimi, Madiha Jamal, Tehreemah Raziq, Zainab Khan, Saed Fawaz Raddawi, Layla Raddaoui, Aamir Omair, Sara Khalid AlNasser, Yara Obaeda Alsouss, Hala Tamim, Khadijah Ateq

**Affiliations:** 1 Department of Biostatistics, Epidemiology and Public Health, College of Medicine, Alfaisal University, Riyadh, Saudi Arabia; 2 Research Unit, College of Medicine, King Saud bin Abdulaziz University for Health Sciences, Riyadh, Saudi Arabia; 3 Communication and swallowing Disorder Division, Prince Sultan Military Medical City, Riyadh, Saudi Arabia; 4 Kinesiology and Health Science, York University, Toronto, ON, Canada; 5 Research Office, King Abdullah International Medical Research Center, King Saud Bin Abdulaziz University for Health Science, Riyadh, Saudi Arabia

**Keywords:** cross sectional study, health education, healthy dietary, nutritional knowledge, obesity, public health, Saudi Arabia

## Abstract

**Background:**

The escalating prevalence of obesity represents a critical public health challenge, especially in Saudi Arabia, where more than 65% of the population is classified as obese. This condition is closely associated with increased mortality and a heightened risk of chronic illnesses such as diabetes and cardiovascular disease.

**Aim:**

This study aims to assess the level of awareness and perceptions regarding healthy dietary choices among individuals in Riyadh, Saudi Arabia.

**Methods:**

A cross-sectional study was conducted in Riyadh, Saudi Arabia, from December 2024 to February 2025, using a convenience sampling method. Adults aged 18 years and older were invited to participate via an online self-administered bilingual questionnaire. The survey assessed knowledge, perceptions, and barriers related to healthy eating. A total of 385 responses were targeted. The questionnaire was adapted from validated tools and an expert checked the questionnaire for clarity and cultural relevance, and suggestions were used to improve it. Internal consistency (reliability) of the final survey items was assessed using Cronbach's alpha, which was 0.6. Descriptive statistics and linear regression analyses were used to explore the associations between sociodemographic factors and knowledge scores. All analyses were performed using SPSS version 29.

**Results:**

After adjusting for confounding variables, being married was significantly associated with higher knowledge of healthy foods (adjusted β = 1.337 [95% CI: 0.097 to 2.576], *p* = 0.035). Arabic speakers displayed significantly lower knowledge of healthy foods than English speakers (Adjusted β = −1.555 [95% CI: −2.318 to −0.792], *p* < 0.001). Additionally, individuals with a bachelor's degree or higher had significantly lower knowledge of healthy foods than those with only a high school diploma (Adjusted β = −0.842 [95% CI: −1.651 to −0.034], *p* = 0.041).

**Conclusion:**

This study highlights notable disparities in dietary knowledge across demographic groups in Riyadh, Saudi Arabia. The findings underscore the need for tailored public health strategies that specifically address young adults, Arabic-speaking populations, and individuals with higher educational attainment, who surprisingly demonstrated lower levels of dietary knowledge. Culturally sensitive, language-specific, and age-appropriate educational interventions are critical to improving awareness of healthy eating habits and ultimately reducing the burden of obesity and related health complications in Saudi society.

**Limitations:**

The study was done in Riyadh, Saudi Arabia, limiting the generalizability of our findings as nutritional knowledge may vary in other regions of the country. Many people find it challenging to eat healthy in Riyadh due to the city's fast-paced culture, with factors such as time constraints, low motivation, cost, and a lack of support all contributing to poor food choices. Moreover, self-reported data in cross-sectional studies can introduce reporting and recall biases. The use of convenience sampling may have led to selection bias, and factors like comorbidities may limit how well the findings apply to the wider population.

## Introduction

As scientific understanding advances, the definition of a nutritious diet continues to evolve, highlighting the complex relationship between foods, nutrients, and overall health. Specific nutrients, food groups, and dietary patterns have been shown to play a vital role in preventing and managing non-communicable diseases (NCDs), including cancer, diabetes, obesity, cardiovascular diseases, cognitive decline, and chronic respiratory conditions—key drivers of global morbidity and mortality. A dietary pattern encompasses the total intake of food and beverages, with each component contributing to health outcomes. As a result, assessing dietary patterns may offer a more comprehensive and accurate prediction of health status and disease risk than evaluating individual nutrients alone (1).

According to the World Health Organization (WHO), a balanced diet should provide sufficient energy while limiting the intake of sugar, salt, saturated fats (such as those found in meat and butter), and trans fats, which are commonly present in fried and processed foods. Instead, it encourages the consumption of unsaturated fats (e.g., those found in fish and nuts), along with a higher intake of fruits and vegetables (2). However, the definition of a healthy diet is not one-size-fits-all; it should be personalized based on an individual's physiological status, age, biochemical and hematological parameters, and lifestyle factors (3).

The rising prevalence of obesity, diabetes, and cardiovascular diseases is driven by a combination of factors, including sedentary lifestyles, increased consumption of fast and processed foods, and inadequate intake of nutrient-dense, whole foods. According to the WHO, global obesity rates among adults have more than doubled since the 1990s, with one in eight individuals classified as obese in 2022 (4). This surge is partly linked to the rapid urbanization of societies, where greater reliance on cars and reduced walkability limit opportunities for daily physical activity (5). Furthermore, socioeconomic disparities and the widespread availability of inexpensive fast food options exacerbate the issue (6, 7).

This relationship between urbanization and obesity extends to several countries, including Saudi Arabia. In addition to urban development, cultural practices also shape dietary habits. Saudi hospitality and social gatherings often involve consuming large quantities of food, leading to increased caloric intake. When combined with a sedentary lifestyle, this highlights a key factor in Saudi Arabia's obesity crisis. Currently, over 65% of the Saudi population has a high body mass index (BMI), contributing to approximately 73% of all deaths, amounting to more than 900,000 fatalities annually (8). Despite various health promotion efforts, including awareness campaigns, the national obesity rate remains at 24.7%, significantly higher than the global average (9). This underscores the importance of regularly assessing public awareness and evaluating the effectiveness of these initiatives.

Compared to other nations, Saudi Arabia possesses a relatively limited understanding of its population's perceptions regarding healthy eating, encompassing knowledge of specific food items, nutritional composition, and food processing methods. As the burden of diet-related diseases continues to rise, there is an urgent need to explore how health education initiatives and public health interventions can more effectively facilitate the adoption of healthier dietary behaviors among Saudi citizens. Despite the implementation of numerous awareness campaigns in recent years, their impact remains insufficiently evaluated. Assessing nutritional literacy across diverse demographic segments—such as age groups, educational levels, and income strata—is essential for informing the design of targeted, culturally relevant strategies. Such an approach will enable the development of more impactful and equitable public health campaigns.

In addition, it is important to identify the barriers that prevent individuals from eating more healthfully. These barriers may include a lack of motivation, limited time, or the belief that healthy food lacks flavor. Understanding these challenges can inform the development of more effective and appealing health programs. By making health messages more relatable and relevant to people's everyday lives, we can foster a more supportive environment that promotes long-term healthy eating habits.

### Aim

This study seeks to comprehensively assess the level of awareness and underlying perceptions related to healthy dietary choices among Saudi Arabian individuals. It aims to explore how cultural, social, and demographic factors influence people's understanding of nutrition, food selection, and overall eating behaviors. The findings are intended to inform the development of targeted public health strategies and educational interventions that promote healthier eating patterns within the Saudi context.

## Methodology

Since our objective was to assess how people in Riyadh, Saudi Arabia, perceive and understand healthy dietary choices, a cross-sectional study was conducted between December 2024 and February 2025 in Riyadh, Saudi Arabia. The target population consisted of adults aged 18 years and older, including Saudi and non-Saudi nationals, currently residing in Riyadh. Eligibility criteria included being 18 years or older, of any gender or nationality, and living in Riyadh. Individuals were excluded if they were under 18 or if they were living outside of Riyadh.

A convenience sampling technique was employed to recruit participants for this study. This non-probability sampling method involves selecting individuals who are readily accessible and willing to participate, making it a practical approach for survey-based research. Data were collected using a structured electronic questionnaire distributed via online platforms. Participants were approached through X, WhatsApp, Facebook, and LinkedIn. Eligibility criteria included adults aged 18 years and older residing in Riyadh. The survey was available in English and Arabic to accommodate linguistic diversity among the target population. Riyadh, Saudi Arabia's population, was estimated at approximately 8 million. Using an online sample size calculator with a 95% confidence level and a 5% margin of error, the minimum required sample size was 385 participants. To account for possible non-responses and improve the reliability of the results, the target sample size was set at 385.

### Questionnaire design

A structured, self-administered questionnaire assessed participants' knowledge, perceptions, and perceived barriers to healthy eating. The questionnaire, adapted from previously validated questionnaires in the U.S. (10) and Saudi Arabia (11), were reviewed for cultural and linguistic relevance. It was made available in English and Arabic to accommodate the primary languages spoken by the target population in Riyadh. Participation was voluntary, anonymous, and limited to individuals 18 years or older. The survey consisted of four sections: (4) demographic data, including age, gender, and monthly income; (12) knowledge of healthy food; (13) access to healthy food; (14) understanding the quantity of healthy food consumption.

The survey began with a demographic section that included questions on gender, age, marital status, educational attainment, and monthly household income. These variables were used to describe the characteristics of the sample and explore potential associations with nutrition knowledge and perceptions. The second section consisted of a series of true/false/I don't know statements designed to assess participants' knowledge of healthy food. These included general nutrition facts (e.g., “The daily nutrient requirement consists of carbs, fats, and protein”), dietary guidelines (e.g., recommended intake levels for sugar, salt, fiber, and fats), and common food-related misconceptions (e.g., “A gluten-free diet is healthy and recommended even for those who do not have gluten sensitivity”). Moreover, 20 questions were included to assess the level of knowledge about healthy food. Each correct response was assigned a value of 1, while each incorrect response was assigned a value of 0. The total knowledge score was calculated by summing the responses, with higher scores indicating greater knowledge of healthy foods. Further questions evaluated understanding of food ingredients and processing methods. Items such as “Fermenting food (e.g., homemade yogurt, kimchi) is healthy” and “Fried chicken nuggets have lower fat content than baked ones” aimed to measure awareness of how food preparation affects health. The third section addressed perceived barriers to healthy eating, such as statements like “Eating healthy food is expensive” and “Healthy food takes more time to prepare,” with participants indicating their level of agreement. The fourth section explored misperceptions about healthy food consumption: “If a food product is healthy, it means I can eat as much of it as I want, whenever I want.”

### Validity and reliability

The questionnaire, adapted from previously validated questionnaires used in nutrition and public health research in the U.S. (10) and Saudi Arabia (11), were reviewed for cultural and linguistic relevance.

To establish face validity, an expert on health sciences reviewed our questionnaire to assess the questions' clarity, ease of understanding, and cultural appropriateness. Feedback was used to review and confirm the suitability of the survey for the local context, improving its effectiveness for the intended audience. Internal consistency (reliability) of the final survey items was assessed using Cronbach's alpha, which was 0.6.

Ethical considerations: Informed consent was obtained from all participants at the start of the online survey, which was conducted voluntarily with the option to withdraw at any point. All responses were anonymized to protect participant identity, and data were stored securely on Google Forms, with access restricted to the research team. The authors sought approval from the Institutional Review Board of Alfaisal University.

#### Data analysis

Descriptive statistics were used to summarize participants' characteristics, knowledge of healthy foods, and attitudes toward food. Simple linear regression analysis was conducted to examine the bivariate associations between each sociodemographic factor and knowledge of healthy foods.

The process used to select confounding variables for the regression model was informed by previous literature identifying factors associated with knowledge of healthy food (such as gender, age, marital status, education, language, and income). Variables that were consistently reported as relevant determinants in earlier studies were included in the model to reduce potential confounding.

Multiple linear regression models were performed to assess the independent associations, including all relevant sociodemographic variables. Unstandardized beta coefficients and their corresponding 95% confidence intervals (CIs) were reported for all regression analyses. Statistical significance was determined at a two-sided alpha level of 0.05. All analyses were conducted using SPSS version 29.

## Results

**Figure 1:**
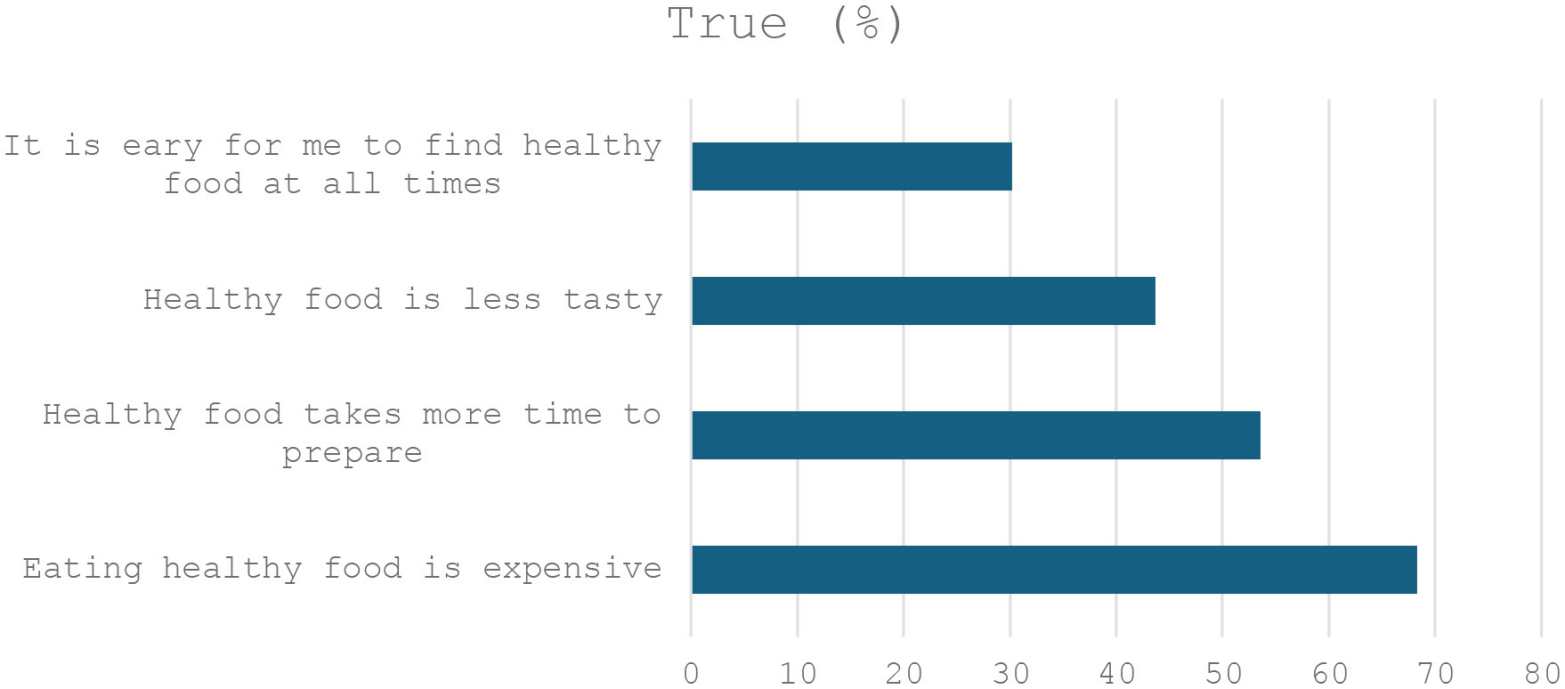
Participants' attitude toward food.

Figure 1 summarizes the participants' attitude to healthy food with approximately 70% agreeing that healthy food is expensive.

Table 1 presents the sociodemographic characteristics of the study participants. The majority of respondents were female (67.7%) and young adults aged 18–24 (71.9%). Most participants were single (78.1%) and had a high school degree as their highest educational attainment (59.9%). English was the primary language of most respondents (77.8%), and a substantial proportion reported a high monthly income (>30,000; 44%).

**Table 1:** Results of the descriptive statistics of participants' sociodemographic characteristics.

**Factor**	**Number**	**(%)**
**Socio-demographic characteristics**		
**Gender**		
Male	108	32.3
Female	226	67.7
**Age**		
18–24 (young adults)	240	71.9
≥25 (Adults)	94	28.1
**Marital status**		
Single	261	78.1
Married	73	21.9
**Education level**		
High school degree	200	59.9
Bachelor degree or higher	134	40.1
**Language**		
English	260	77.8
Arabic	74	22.2
**Income**		
Less than 15,000 (Low Income)	114	34.1
15,000–29,999 (Medium Income)	73	21.9
More than 30,000 (High Income)	147	44

Table 2 summarizes the Participants' responses to the healthy food knowledge items. Overall, high awareness was observed for general dietary recommendations, such as daily fruit and vegetable consumption and healthier protein choices. However, notable misconceptions were identified regarding recommended intake levels of macronutrients, sugar, salt, fiber, and fat, as well as food processing methods, indicating gaps in detailed nutritional knowledge.

**Table 2:** Participants' knowledge of healthy foods.

**Knowledge of healthy foods**	**% Correct (n)**	**% Incorrect (n)**
Eating healthy food cannot help prevent chronic medical conditions/diseases (False)	74.9 (250)	25.1 (84)
The daily nutrient requirement consists of carbs, fat and protein (True)	83.5 (279)	16.5 (55)
The maximum intake of trans-fatty acids is 20% of the total diet (False)	13.2 (44)	86.8 (290)
The maximum intake of saturated fat per day is 30% of the total diet (False)	17.4 (58)	82.6 (276)
The maximum intake of carbs per day is 60% of the total diet (False)	22.2 (74)	77.8 (260)
The maximum intake of protein per day is 10% of the total diet (False)	37.4 (125)	62.6 (209)
The nutrient content of the food (amount of fat, protein, and carb) is enough for me to decide if a food is healthy (False)	40.1 (134)	59.9 (200)
A gluten-free diet is healthy and recommended even for those who do not have gluten sensitivity (False)	46.4 (155)	53.6 (179)
Fruits and vegetables should be eaten daily (True)	94 (314)	6 (20)
White bread has more fiber than brown bread (False)	73.4 (245)	26.6 (89)
Chicken and fish meat are healthier than red meat (beef) (True)	67.7 (226)	32.3 (108)
Butter increases cholesterol more than vegetable oil (True)	46.4 (155)	53.6 (179)
If the same quantity of chips and nuts are eaten, chips are a healthier than nuts (False)	72.2 (241)	27.8 (93)
The minimum intake of fiber per day is 25 g (True)	27.2 (91)	72.8 (243)
The minimum intake of sugar per day is 2 g (True)	24.3 (81)	75.7 (253)
The maximum intake of salt per day is less than 5 g per day (True)	32.9 (110)	67.1 (224)
Unsaturated fats are healthier than saturated fats (True)	58.1 (194)	41.9 (140)
Barbecuing food is not healthy (True)	25.7 (86)	74.3 (248)
Canned food lose all their nutritional value (False)	35.3 (118)	64.7 (216)
Fermenting food (homemade yogurt, kimichi) is healthy (True)	76.6 (256)	23.4 (78)

Table 3 presents the results of the bivariate and multivariate regression analyses examining the relationship between sociodemographic variables and knowledge of healthy foods. At the bivariate level, adults (≥25) exhibited significantly higher knowledge of healthy foods compared to young adults aged 18–24 (UnAdj β = 0.744 [95% CI: 0.045 to 1.444], *p* = 0.037). Similarly, married individuals demonstrated significantly higher knowledge than their single counterparts (UnAdj β = 1.328 [95% CI: 0.575 to 2.080], *P* < 0.001). Arabic speakers had significantly lower knowledge of healthy foods compared to English speakers (UnAdj β = −1.735 [95% CI: −2.474 to −0.996], *p* < 0.001), and no significant differences were found in gender, education level, or income. After adjusting for all variables in the multivariate analysis, being married remained significantly associated with higher knowledge of healthy foods (Adj β = 1.337 [95% CI: 0.097 to 2.576], *p* = 0.035). Arabic speakers continued to show significantly lower knowledge of healthy foods than English speakers (Adj β = −1.555 [95% CI: −2.318 to −0.792], *p* < 0.001). Individuals with a bachelor's degree or higher had significantly lower knowledge of healthy foods compared to those with only a high school diploma (Adj β = −0.842, [95% CI: −1.651 to −0.034], *p* = 0.041). Lastly, no significant associations were found between gender, age, or income and knowledge of healthy foods.

**Table 3:** Results of the bivariate and multivariate regression analyses examining the association between sociodemographic factors and knowledge of healthy foods.

**Factor**	**Knowledge of healthy foods**					
	**Unadjusted** β **(SE)**	***p-*** **value**	**95% CI**	**Adjusted** β **(SE)**	***p*** **-value**	**95% CI**
**Socio-demographic characteristics**						
**Gender**						
Male	Ref			Ref		
Female	−0.132 (0.344)	0.702	−0.809, 0.545	−0.073 (0.342)	0.832	−0.746, 0.601
**Age**						
18–24 (young adults)	Ref			Ref		
≥25 (adults)	0.744 (0.356)	0.037	0.045, 1.444	0.389 (0.636)	0.542	−0.863, 1.640
**Marital status**						
Single	Ref			Ref		
Married	1.328 (0.383)	< 0.001	0.575, 2.080	1.337 (0.630)	0.035	0.097, 2.576
**Education level**						
High school degree	Ref			Ref		
Bachelor degree or higher	−0.253 (0.328)	0.442	−0.898, 0.393	−0.842 (0.411)	0.041	−1.651, −0.034
**Language**						
English	Ref			Ref		
Arabic	−1.735 (0.376)	< 0.001	−2.474, −0.996	−1.555 (0.388)	< 0.001	−2.318, −0.792
**Income**						
Less than 15,000 (low income)	Ref			Ref		
15,000–29,999 (medium income)	0.172 (0.440)	0.697	−0.694, 1.037	0.326 (0.423)	0.442	−0.507, 1.159
More than 30,000 (high income)	−0.434 (0.366)	0.237	−1.155, 0.287	−0.311 (0.354)	0.380	−1.007, 0.385

## Discussion

This study identified notable sociodemographic disparities in nutritional knowledge among adults residing in Riyadh. Contrary to expectations, participants with bachelor's degrees or higher demonstrated significantly lower knowledge of healthy foods than those with only a high school education. This finding diverges from prior research in Saudi Arabia by Ayad et al. (15), which reported higher awareness levels among individuals with postgraduate qualifications (master's and Ph.D. degrees) relative to those with lower educational attainment. International research has also shown a similar pattern of association between increased nutritional awareness and higher education. A large cross-sectional research study in Wuhan, China (*n* = 33,436), and another in Quebec, Canada (*n* = 1,902), both indicated a positive association between higher educational attainment and enhanced nutritional awareness (16, 17). Similarly, a study in Jordan with individuals aged 60 and older (*n* = 1,200) revealed significantly decreased knowledge levels among those with limited formal education (18). One possible explanation for this discrepancy may lie in differences in sample composition and survey design. While the present study included a more diverse population from various educational backgrounds and relied on a detailed assessment tool adapted for local relevance, the earlier study had a narrower sample and potentially different measurement criteria. Additionally, it is plausible that individuals with higher academic degrees may not necessarily have received formal education or training in nutrition or health-related topics, which could explain their lower performance in nutrition-specific assessments (19).

Although there is limited evidence directly linking higher education to reduced nutritional knowledge, the association between education level and nutritional knowledge is complex.

A cross-sectional study, carried out in Quebec, Canada, found a significant relation between nutritional knowledge and diet quality among people with lower education but not among those with higher education. This suggests that higher educated people do not rely on nutritional knowledge as much as people with lower education do (17). Another research found that higher education can influence eating habits through a phenomenon called cultural capital, which is defined as the skills, habits, and awareness people collect throughout life (20). Cultural capital explains how education shapes the environments and social circles people are a part of, and if exposed to healthier food lifestyles, they will eventually adopt them as a habit and a norm of experience. This can explain why individuals with higher education in our study have a lower knowledge score. Due to the cultural capital effect, more educated people make healthier food choices even if they cannot recall nutritional facts. So, their lower scores might not mean they lack the knowledge of healthy food, but that they use a different way of making healthy food choices.

Married participants in our study scored significantly higher on nutrition knowledge than their single counterparts. Two recent studies carried out in Saudi Arabia supported our findings. The first study evaluated mothers' comprehension of nutritional standards and portion sizes, including MyPlate. The results indicated that 29.1% of moms recognized MyPlate standards, whereas 51.5% were influenced by healthy plate selections (21). The second study, conducted in Tabuk, revealed that married participants often exhibit improved dietary choices, primarily due to the shared responsibility of their spouses, who tend to share health goals and promote healthier behaviors, consequently promoting a healthier lifestyle (11).

Cross-sectional studies in Sri Lanka (*n* = 400, including 328 married women), and in Edirne, Turkey (*n* = 380, including 131 married women), consistently demonstrated that married women exhibited higher average nutritional knowledge scores compared to their unmarried counterparts (22, 23). Similarly, a study from Quebec, Canada, involving 1,902 adults, revealed that married individuals scored higher in nutritional knowledge assessments (23). Conversely, a study performed in the United States showed a disruption in this consistency. It examined the influence of marital status on the understanding of nutrition and food categories, classifying individuals as single, divorced, widowed, married, or living with a partner. The findings indicated no significant disparity in dietary habits across these groups. This may result from varying cultural norms in Western civilization that influence individuals' approaches to healthy eating (24).

Another notable finding was that Arabic speakers in Saudi Arabia have significantly lower knowledge of healthy foods than English speakers. Limited research has assessed the differences in food habits and perceptions of healthy eating between Arabic-speaking and English-speaking communities in Saudi Arabia, suggesting the need for further research. However, studies in the Middle East provided some insight into this issue. Despite a shift toward global healthy trends, traditional practices remain prevalent in Arabic-speaking communities (25). Conversely, English-speaking communities often combine international flavors with local foods. The young and urban populations in these regions are more inclined to adopt global dietary trends, such as plant-based diets, motivated by health considerations rather than environmental concerns (26).

Cultural and language differences also have a significant effect on how nutritional knowledge is shared between Arabic- and English-speaking communities. Studies show that even highly educated Saudi students face translation errors, especially because they do not know the different social and cultural contexts of the language like native speakers do. This is supported by a study done by Ababneh (27), which found many translation mistakes in basic areas like word choice and sentence structure due to cultural factors. Alqahtani (28) found that translation students had more technical language skills, such as language rules and structure, but lacked the cultural understanding necessary to correctly convey information from English resources. Therefore, while English-speaking Saudis have direct access to international nutritional information and online awareness campaigns, Arabic-speaking Saudis receive translated versions that may convey different meanings.

Our study found no statistically significant difference in nutritional awareness between females and males. This finding contrasts with multiple previous studies within Saudi Arabia and internationally, which consistently reported higher nutrition knowledge scores among females. For instance, studies by Alfawaz et al. (29) and Bookari (30) in Saudi Arabia observed significantly greater nutritional awareness among women. Similarly, Svendsen et al. (31) reported that female university students and employees in Norway demonstrated higher levels of functional nutrition literacy, particularly about dietary guidelines. Comparable gender-based disparities were also observed in studies from Turkey and Pakistan, where females consistently outperformed males in understanding nutritional concepts (32, 33).

The discrepancy between our findings and the existing literature may be attributed to several factors. First, the sample size in our study, though sufficient for overall analysis, may not have been large enough to detect subtle differences across gender subgroups. Second, the dissemination and accessibility of health information through digital platforms may have reduced the historical gap in nutritional awareness between males and females, particularly among younger populations. Lastly, cultural shifts in health engagement and educational access may contribute to narrowing gender differences in this domain. This trend warrants further investigation through longitudinal and nationally representative studies.

Although our study revealed no association between income and nutrition knowledge, several studies have demonstrated a significant relationship between income levels and dietary quality and nutrition knowledge. A study among Saudi adults evaluating knowledge of nutrient-dense foods revealed that higher income significantly predicts better nutrition knowledge scores (34). Similarly, studies conducted across multiple provinces in Central China and Pakistan have found that higher household income was positively associated with increased nutrient consumption (35, 36). A cross-sectional study involving 1,092 adults in the province of Quebec, Canada, reported that individuals with higher income levels scored significantly higher in nutrition knowledge assessments (17). Furthermore, a review of eight studies conducted in Brazil revealed that lower-income individuals were more likely to make unhealthy food choices, even after controlling for cost and accessibility (37). The difference in results between our study and the literature may be attributed to our smaller sample size.

## Limitations and strengths

The results should be interpreted considering several limitations. First, reliance on self-reported data in cross-sectional research may lead to reporting and recall biases. Second, dependence on convenience sampling may have introduced selection bias, confounding bias such as comorbidities, consequently limiting the generalizability of the findings to the broader community. Thirdly, since this study was done in Riyadh, the results have limited generalizability as food habits can vary in other parts of Saudi Arabia. Riyadh is an urban city, and life is fast-paced; many studies show that lifestyle plays a major role in poor dietary habits in Riyadh. Aldosari et al. (12) found that many people in Riyadh find it difficult to make healthy food choices, mainly because they lack the time and willpower. Other factors include less access to healthy food options, lack of family support in making healthy meals, low motivation and cost (13, 14).

Despite these limitations, this study possesses several essential strengths. It is the first study in Saudi Arabia to compare Arabic-speaking and English-speaking communities and assess the impact of primary language on nutritional knowledge. An inclusive assessment was accomplished by utilizing a bilingual, culturally relevant survey tool, revealing that Arabic speakers exhibited significantly lower nutrition knowledge. The findings underscore the importance of including language and cultural context in future regional public health initiatives and studies.

## Conclusion

This study highlights notable disparities in dietary knowledge across demographic groups in Riyadh, Saudi Arabia. The findings underscore the need for tailored public health strategies that specifically address young adults, Arabic-speaking populations, and individuals with higher educational attainment, who surprisingly demonstrated lower levels of dietary knowledge. Culturally sensitive, language-specific, and age-appropriate educational interventions are critical to improving awareness of healthy eating habits and ultimately reducing the burden of obesity and related chronic health conditions in Saudi society.

Our findings suggest that while the overall population has some awareness of healthy food options, significant disparities persist across specific subgroups. Nutritional knowledge was shown to be positively associated with marital status; married individuals consistently outscored their single counterparts. Notably, those with higher education levels and Arabic speakers had inadequate nutrition knowledge, necessitating further investigation into the accessibility and content of health education across various languages and educational environments. The unexpected result showing that individuals with higher education had lower dietary knowledge may indicate a gap between academic achievement and practical health literacy. Possible explanations include the lack of nutrition-related content in higher education programs, greater exposure to inaccurate information through digital and social media channels, and lifestyle pressures such as limited time, work-related stress, and dependence on convenience foods.

Understanding these demographic factors, particularly for unmarried individuals, Arabic speakers, young adults aged 18–24, and those pursuing higher education, is crucial for developing effective interventions. Future initiatives should focus on integrating nutrition education into university curricula and expanding outreach programs tailored to the linguistic and cultural diversity of the Saudi population.

## Future recommendations

Given that most research on this subject in Saudi Arabia is cross-sectional, further studies have to use longitudinal and interventional designs to evaluate the enduring effects of nutrition education on dietary practices and attitudes. To enhance generalizability, including nationally representative samples encompassing a broader range of demographic and socioeconomic categories is essential.

Further investigation is needed to understand why individuals with higher education levels have lower dietary knowledge, which may reflect limited nutrition education or the absence of mandatory health courses in college curricula. Similarly, our findings that Arabic speakers have less nutrition information than English speakers highlight the need to examine how primary language and cultural background influence dietary knowledge and habits. To address these gaps, public health efforts should focus on targeted nutrition programs in university courses and community initiatives for young adults, Arabic speakers, and diverse populations.

Providing reliable Arabic-language resources and using social media to share evidence-based information can help counter misinformation. Involving healthcare providers, educators, and community leaders in designing and delivering these programs will make them more practical, effective, and sustainable in the Saudi context.

Finally, our research defined “healthy food” by WHO guidelines. Future qualitative research may investigate whether individuals perceive these global standards to be relevant and whether local context or customer feedback should inform subsequent health guidelines.

## Data Availability

The raw data supporting the conclusions of this article will be made available by the authors, without undue reservation.
